# Evaluating Nurse Conscientious Objection: Application of a Novel Framework

**DOI:** 10.1007/s10730-025-09556-7

**Published:** 2025-08-09

**Authors:** Maya Zumstein-Shaha, Lucia D. Wocial, Vicki D. Lachman, Norah Louise Johnson, Cynda Hylton Rushton, Pamela J. Grace

**Affiliations:** 1 Department of Health, Bern University of Applied Sciences Health, Murtenstrasse 10, Bern, CH-3008 Switzerland; 2 Faculty of Health, Department of Nursing, University of Witten/Herdecke, Witten, Germany; 3 Faculty of Health, Bournemouth University, Poole, UK; 4 John J. Lynch Center for Ethics, MedStar Washington Hospital Center (MWHC), Washington, DC USA; 5 V.L. Associates, Sarasota, USA; 6 College of Nursing, Marquette University, Milwaukee, USA; 7 Johns Hopkins University School of Nursing & Berman Institute of Bioethics, Baltimore, USA; 8 Boston College, Chestnut Hill, USA

**Keywords:** Conscientious objection, Conscientious commitment, Palliative sedation, Framework for evaluation conscientious objection, Visitor restriction

## Abstract

Certain moral beliefs and/or values about what is good or harmful can cause nurses and other healthcare professionals to object to participating in some clinical actions. Such objections are also called conscientious objections. Invocation of a conscientious objection (CO) can produce complexities in patient care and health care delivery and must be mindfully evaluated for its soundness. In this manuscript, a recently developed framework, The Ethical Evaluation of a Nurse’s Conscientious Objection (EENCO), is applied to expose hidden elements and nuances in a proposed or actual CO by nurses or other healthcare professionals, thereby illuminating strategies that can lessen associated harms. The EENCO is utilized to explore two types of situations where a nurse makes a CO claim. Scenario 1 involves a nurse’s reluctance to follow provider medication orders intended to relieve pain and suffering at the end-of-life. In scenario 2, nurses object to a visitation policy during the COVID-19 pandemic. Additionally, we provide a summary of the necessary elements of institutional policy to address claims of CO using the EENCO. Drawing on the EENCO, the two scenarios were analyzed for their ethical implications. This framework contributes to the exposure, scrutiny, and clarification of potentially unappreciated aspects of CO claims. Steps for developing institutional policy are identified. Application of the EENCO guides the analysis of the two scenarios. CO claims are explored more deeply, thereby revealing implications for those involved. Additionally, the EENCO provides guidance for the development of institutional CO policies.

## Introduction

Evaluating claims of conscientious objection (CO) in healthcare is inevitably an ethical undertaking because unexamined they can result in a nurse or other healthcare professional (HCP) refusing to participate in a legally available treatment based on their personal values or biases. While we largely focus on the responsibilities of professional nurses here, extrapolations can be made to the responsibilities of other healthcare professionals who may be asked to participate in actions that are in opposition to their personal values. What is legally sanctioned, of course, varies from state to state in the United States of America (USA) and among countries, and legal considerations are relevant to decision-making about CO. However, when a legally supported service, such as giving adequate pain medicine, is the subject of a CO, patients may not receive the recommended or desired treatment in a timely manner, and consequently be unnecessarily harmed or their care rendered suboptimal (Charo, 2005; de Londras et al., 2023; Fry-Bowers, 2020; Savulescu, 2006). We exemplify these points later, using supporting evidence to highlight that the most vulnerable to their health and circumstances may be disproportionately at risk (Grace et al., 2024). To remedy the situation other clinicians may have to assume associated patient care responsibilities creating disproportionate or inequitable burdens on certain members of the healthcare team. While the implications of making a claim of CO to involvement in a proposed or actual action apply to all HCPs, in this paper we focus on nurses for two reasons. Globally, nurses constitute the largest number of healthcare personnel by far (Boniol et al., 2022). We see CO as including both a nurse’s personal morally-based objection to being involved in an action or proposed action AND a nurse’s objection to the actions of others that the nurse views as likely harmful to patients or others. They both entail objecting to participation in an action based on what has been loosely referred to as conscience but more helpfully defined as deeply held values and beliefs (Wicclair, 2011).

Despite well-argued ethical and legal defenses of healthcare providers’ rights to make a CO, a risk remains that the individual claim can be considered arbitrary, not well thought-out, or biased by those evaluating and who in turn may exhibit similar biases toward the person claiming a CO (Grace et al., 2024). Thus, care must be taken to understand the basis of a person’s CO related to its authenticity. By authenticity, we mean a reflected-upon exploration of the presenting situation, an ability to describe why and how it is disturbing, its relevance to one’s deeply held values and beliefs, and an ability to demonstrate an understanding of its likely impact on the patient and others involved. In a prior article, we acknowledged the need for those involved in an actual or potential situation of CO to evaluate circumstances with humility.

The virtue of humility in CO involves embracing the diverse experiences of patients and staff. Humility is the quality of being modest, respectful, and involves recognizing one’s limitations, valuing others’ perspectives, and fostering a willingness to learn rather than asserting superiority (Michalec et al., 2024). It involves an open-mindedness to learn from experiences and a willingness to acknowledge mistakes without excessive pride or defensiveness (Wadhwa & Mahant, 2022). Humility fosters better relationships by promoting empathy; therefore, it allows individuals to acknowledge the complexities of the moral aspects underlying a CO. By approaching their role with humility, nurses ensure that their objections are seen as thoughtful, rather than divisive or self-righteous. For example, a nurse could state, “I understand why you might see it that way, and here’s why I see it differently.” A statement such as this one allows the nurse to acknowledge what someone else believes as well as acknowledge their own beliefs. Likewise, those charged with evaluating or managing a CO, or designing policy also need to do so with humility.

It is also clear that nurses must be supported when a claim of CO is grounded in deeply held and defensible personal and/or professional values (American Nurses Association, 2025; ICN International Council of Nurse, 2021). This is an ethical right derived from the idea of human rights and that one does not give up one’s human rights on entering a profession (American Nurses Association, 2015, Provision 5; Grace & Welch, 2023). Nurses should be free to object to participating in actions they find morally indefensible (personally or professionally), and are legally supported in many but not all countries (Fiala & Arthur, 2017). Alternatively, nurses along with other HCPs may be asked to participate in actions or follow protocols that will not benefit and will likely harm patients or others. As noted in nursing literature and nursing codes of ethics, there is a professional obligation to object to such proposals and actions that cannot further nursing goals and may harm people.

Honoring a nurse’s CO almost always has some negative consequences for the nurse, patient, and/or others. Two common examples with different underlying motivations are: (1) a nurse is excused from providing patient care because of deeply held values and beliefs that the action is morally wrong, even though legally permissible, in which case others must fill the care gap, or (2) a nurse intervenes, based on professional values, to prevent visibly impaired colleague or allied team member from engaging in an invasive intervention which they are obviously unable to complete safely. All who provide direct patient services, regardless of profession, face similar issues. The question we address is “how can a claim of conscientious objection be evaluated for its soundness?” To reiterate, this exploration while focusing on nurses and the nursing profession is applicable to other HCPs.

Recently, a group of nurse ethicists developed a framework for Ethical Evaluation of a Nurse’s Conscientious Objection (EENCO) to guide analysis and evaluation of CO claims made by nurses (Grace et al., 2024). The main reasons for this were the need to provide guidance for those charged with analyzing and evaluating claims of CO. In addition, an extensive search of the literature failed to identify a framework that is specific to the purpose of evaluating the various types of CO that occur in nursing settings (Grace et al., 2024). Subsequently, two types of CO are explored below using the EENCO framework. One concerns the situation of an individual nurse expected to participate in an action that seems morally disturbing to them. The second is a case exemplifying the problem of nurses being complicit in harms or potential harms caused by others, regulations, or protocols. The purpose of this manuscript is to demonstrate the application of the EENCO framework to situations where clarification of the ethical status of a CO is needed and next steps are identified. Finally, we offer suggestions for unit or institutional policy development related to CO.

## Evaluation of Conscientious Objection: Difficulties

As noted earlier, the right to CO in healthcare settings is protected by some state and federal laws in the United States of America (USA) (Department of Health and Human Services, 2024) and in the laws of many countries. However, legal protections do not exempt the person from non-legal consequences or accountability for associated professional responsibilities (Fry-Bowers, 2020). The literature is replete with stories about nurses’ struggles to determine when a CO to a proposed action, to the actions of others, or to a policy is required, and what that means for them. In turn leaders, administrators, and policy makers seek clarity about how to evaluate a claim of CO, when to support the claim, how, when sanctions are appropriate, and what sorts of sanctions are required. Two important categories of CO in healthcare are those of a conflict of conscience (Wicclair, 2017), and a conflict of commitment (Dickens & Cook, 2011). In either case, a decision must be made about how to address these claims of CO that put nurses at risk from retaliation or unrelieved moral distress (Grace et al., 2024) and patients at risk of not having their needs met at minimum and at worst from being harmed.

Compounding the problem of CO is that the ability to exercise a legitimate claim may be influenced by the power imbalances that often exist in healthcare, exacerbating the potential for harms (Hart, 2015; Okpala, 2021). Thus, exploring the implications of an actual or potential CO claim needs to be conducted carefully to anticipate, alleviate or manage possible sequelae (Grace et al., 2024). Additionally, evidence points to the problem that COs to participating in certain interventions, such as abortion care, tend to most disadvantage patients who have limited options to receive the care for which they legitimately have a right (Fry-Bowers, 2020). Alternatively, nurses who are objecting to what they see as the unethical practice or directives of others are also disproportionately at personal risk because of the hierarchical nature of healthcare delivery, and expectations that they follow orders of those “above” them. Depending on where they practice, their actions and choices may have legal consequences (Fry-Bowers, 2020).

Another difficulty is that the meaning of conscience as a concept remains contested and multiple definitions exist (Pilkington, 2021). Here, we rely on Wicclair’s (2011) argument that in discussing CO we are really looking at “an important subset of an agent’s ethical or religious beliefs– *core* beliefs” (italics as in the original) (p. 4). Thus, objections to participation in healthcare associated actions based on conscience are amenable to exploration and must be examined carefully to ensure objections do not arbitrarily disadvantage persons. As argued by Grace and colleagues (2024), clarity about the scope, limits, and circumstance in which CO may be unsupportable, permissible, or imperative has been elusive. This elusiveness is problematic for analyzing, evaluating, supporting, and/or sanctioning individuals who claim CO and for the development of guiding policies - educational, institutional, and societal (regulatory). Yet, the American Nurses Association (ANA Center for Ethics and Human Rights, 2022), among other professional organizations, highlights the responsibilities of organizational policies related to CO. They note that “To manage risks and protections, healthcare organizations should have policies and procedures, which address such items” (ANA Center for Ethics and Human Rights, 2022, p. 3). Healthcare institutions have obligations to lessen harm to patients while protecting clinicians.

We propose the EENCO framework as a reasonable way to analyze and evaluate CO claims and to determine appropriate support and sanctions across levels: individual, supervisory, and institutional. Additionally, those charged with educating nurses and other HCPs can use this framework to help develop reflective practitioners who understand their professional responsibilities to patients.

## Using the Framework* Ethical Evaluation of a Nurse’s Conscientious Objection (EENCO)*

This framework emerged as the result of both a comprehensive literature review and discussions among a group of nurses with ethics expertise. It consists of considerations and questions to address claims of CO, which were presented in table-form in the prior article (Grace et al., 2024, pp. 252–253). The table is arranged according to the psychologist James Rest’s four components of cognition underlying moral action, namely *moral sensitivity*, *moral judgment*, *moral motivation,* and *moral character and implementation* (Grace et al., 2024). Rest’s theory provides an explanation of the psychological processes underlying ethical decision-making and is based on research in moral and cognitive psychology. “Both cognition and affect are intertwined in each component” (Narvaez & Rest, 1995, p. 385) and are inseparable. In this sense, cognition is not the same as pure rationality or the ability to reason unaffected by emotion or other influences. The components explain both cognitive and emotive aspects of human decision-making (Rest, 1982). They are postulated to work interactively when a person is trying to determine how to enact a “good” in problematic circumstances (Grace et al., 2024). Moral sensitivity is about appreciating the ethical nature of a situation; whereas moral judgment is the process of deliberation about a good action and includes identifying and acquiring necessary information. Moral motivation has both cognitive and emotive aspects and is about the impetus to act based on the chosen option. Finally, moral character and implementation have cognitive and emotive aspects related to the skills needed to persevere in carrying out the chosen action (Grace et al., 2024).

To demonstrate the use of the framework “EENCO”, two common types of CO scenarios using the perspectives of both the objector(s) and an evaluator are presented. The first scenario involves an individual nurse who is reluctant to participate in a legitimate and ethically recommended process of palliative sedation. The second scenario also involves nurses who bore witness to and felt complicit in the harmful consequences to patients and families of a crisis standard initiated during the COVID-19 pandemic. Application of the EENCO to claims of CO requires selecting the questions most applicable and considerations depending on the relevance and context of a given situation. For current purposes, we used five essential questions from the EENCO that reflect Rest’s model and the literature (Grace et al., 2024). The questions are:

1. How does the nurse understand their professional responsibility and its limits? (moral sensitivity)
2. What does it mean to say a conviction is held strongly enough that the likely harms to the objector outweigh the likely good for the patient? (moral judgment)
3. What strategies and resources are available to assist in exploring a particular situation where the nurse is considering or executing a CO? (moral judgment)
4. What are the nurse’s, leaders’, and institution’s responsibilities to ensure that a patient has their needs met if the CO is supported? (moral motivation and moral character)
5. What institutional or leadership support is available for nurses with respect to CO? (moral judgment and moral character applied to unit and institutional leadership) (Grace et al., 2024, p. 251).

These questions are predominantly explored from the perspective of the charge nurse in case 1 and from the perspective of the nurses in case 2. However anyone charged with helping to resolve a situation of CO in healthcare could use these to explore the situation.

### Case 1– Conscientious Objection To Palliative Sedation

This is a composite case, names are fictitious. Derinda Hayek is a recently graduated nurse, hired six months ago to work on the medical oncology unit of a safety net hospital serving a metropolitan area in the USA. Until this week, she had been assigned a mentor to help her adjust to independently caring for a group of patients.

As part of her orientation for her current position, Derinda had several discussions with unit leaders on the organization’s mission and values, unit processes, and procedures. The leaders discussed with Derinda the patient population served on the unit, their needs, and the most important treatments provided on this unit. The unit leaders specifically addressed pain management procedures at the end of life such as palliative sedation. The main purpose of this extensive orientation was for leaders to determine any concerns that new nurses might have regarding the unit’s core practices. At the same time, the unit leaders also clarified the expectations of nurses on the unit. The orientation concluded with the leader reviewing the content with Derinda who verbalized key actions central to her role and responsibilities. Derinda acknowledged the importance of pain management in mitigating the symptoms of cancer and its treatment, admitted to her lack of experience in this area, and expressed an eagerness to learn.

Today, Derinda is caring for a 51-year-old woman, Sandra, diagnosed with stage IV uterine cancer that has infiltrated sacral and other nerves, causing refractory pain while she is awake despite major increases in narcotics. Now assessed to be imminently dying, the medical team had asked the palliative care service to evaluate Sandra for ways to mitigate her refractory pain. Three days prior, the team of a palliative care physician and nurse practitioner talked at length with Sandra and her husband who she has legally designated as her proxy-decision maker in the event she loses decision-making capacity. Several strategies for pain management were discussed, including increasing doses of narcotics, targeted nerve blocks to prevent pain messages from the cancer infiltration reaching her brain and palliative sedation. Palliative sedation is “… the monitored use of medications intended to induce a state of decreased or absent awareness (unconsciousness) to relieve the burden of otherwise intractable suffering” (Beauverd et al., 2024, p. 3590). The patient may remain in a coma until their death if symptoms cannot be relieved otherwise. It was made clear that some of these interventions could shorten Sandra’s life. Regardless. Sandra indicated that “death cannot be worse than this pain,” and “I don’t have long anyway.”

The team requested an ethics consultation to evaluate the permissibility of palliative sedation for Sandra. The resulting recommendation concluded that due to the refractory nature of her intractable pain, her imminent death, and her appreciation of the meaning of the process of palliative sedation, this procedure was ethically permissible. They suggested the team provide support for the person who would need to assume decision-making during the procedure of palliative sedation (as the patient would be rendered unable to speak for herself).

The palliative sedation regimen is scheduled to begin this morning. After hearing the report, Derinda tells the charge nurse that although she does understand the patient’s need for pain control, her religious beliefs about the sacredness of life are strong and actions that foreshorten life are a violation of her core beliefs. She requests a change of assignment.

#### Analysis of Case 1

Frameworks for ethical analysis consist of considerations and are not linear in application. As information is gained, understanding of nuances of the situation unfold, often leading to new questions. However, a good starting place for the charge nurse is to explore Derinda’s concerns and her understanding of her professional responsibilities to the patient.

1. How does Derinda understand her professional responsibility and its limits from the perspective of the charge nurse? (Rest’s Moral Sensitivity) (Grace et al., 2024, p. 251).

The charge nurse or unit leader, responsible both for evaluating the claim and supporting those involved, is faced with a challenging situation. First, she must appreciate the ethical nature of the situation by determining whether Derinda is objecting because she feels insufficiently prepared or she has genuine qualms about the idea of palliative sedation. Here, it is disclosed that Derinda is objecting on religiously informed moral grounds. She feels that participating in palliative sedation goes against her religious beliefs regarding the sacredness of life. Therefore, Derinda is prohibited in participating in acts that hasten death. From her perspective, palliative sedation not only would eliminate the patient’s awareness but also potentially hasten her death. It is also possible that Derinda feels inadequately prepared to care for the patient. In either case, further clarification is needed.

Sandra is in great distress due to refractory pain from end-stage cancer. Her pain is unbearable, and palliative sedation is the treatment she has chosen to relieve her pain. She has decision-making capacity, and her decision is informed at the time she made it. Sandra’s husband, now her proxy decision-maker, is supportive of her request. He does not want to lose her but agrees this is in her interests. It is hard for him to bear witness to her suffering.

The nurse, Derinda, who is making the CO, has deep seated beliefs that prohibit her from participating in the palliative sedation. In contrast, the patient needs nursing care. It is likely that work assignments need to be rearranged within the nursing team to address Derinda’s CO. Ethical sensitivity means Derinda understands both her professional obligations to provide a “good” or benefit for Sandra including relieving her suffering, and concerns about reconciling this with a conflicting belief that she would be doing harm by participating in palliative sedation. Factoring into the charge nurse’s decision are the consequences of honoring Derinda’s request, including how others may be affected by this situation. After the immediate situation is addressed, the charge nurse, or unit leader, will need to help Derinda identify how her ultimate decision may affect others in the environment, as discussed shortly.

The charge nurse must work with Derinda to explore the ethical implications of her proposed inaction and provide guidance. Leadership action in such instance includes trying to understand but not discount Derinda’s beliefs. Similarly, Derinda needs to be able to cite her reasons for not taking part in palliative sedation. The analysis of this case and clarification of professional responsibilities may help Derinda to be clearer about whether her refusal of participation is permissible based on her conscience. The following are considerations for the charge nurse.

- Derinda’s prior preparation during her basic nursing education (from the perspective of the charge nurse).

Ideally, opportunities to explore similar situations, and gain some confidence in ethical decision-making, would have occurred during basic nursing education. Critical to nursing practice is the idea that it is trust-based and involves responsibilities to balance risk to patient with risk to self, judiciously prioritizing patient needs over one’s own except when the risk to self is (too) high. Both the American Association of Colleges of Nursing (AACN) (2021) and the International Council of Nursing (ICN) (2021) stress the importance of nurses’ understanding their professional responsibilities. “The development of a nursing identity embracing the values of integrity, altruism, inclusivity, compassion, courage, humility, advocacy, caring, autonomy, humanity, and social justice” (AACN, 2021, p. 49; Crigger & Godfrey, 2014). The first provision of the ANA code of ethics (2025) asserts that nurses must treat every person with respect including respect for choices that are different than their own. Additionally, “(N)urses have an ethical responsibility to relieve pain and the suffering it causes” (ANA Center for Ethics and Human Rights, 2022, p. 1). Acts such as palliative sedation can be ethically permissible when certain conditions are met.

b) Drawing on the doctrine of “Double Effect” (DDE).

This doctrine can help to distinguish between intended effects and those that are foreseen but unintended (McIntyre, 2001). While not without its critiques including that “intention” is hard (if not impossible) to validate, it can help those - perhaps especially those with strong religious beliefs - to provide adequate pain relief even in the face of hastened death. Indeed, its origin in Roman Catholic thinking dating from Augustine was to provide a way to justify killing in self-defense (McIntyre, 2023). One’s intent is not to kill the other person but to save oneself from being killed. With this perspective, palliative sedation is viewed as a means that leads to the alleviation of refractory pain at the end of life. As such, the risk for hastened death is unintended and, therefore, palliative sedation is permissible (Faris et al., 2021).

Despite the potential preparation in nursing education and this argumentation, Derinda’s fears may not be allayed. Those without strong religious beliefs who nevertheless cannot reconcile participation with their sense of integrity should also be supported.

2. What does it mean to say a conviction is held strongly enough that the likely harm to the objector outweighs the likely good for the patient from the perspective of the charge nurse? (Rest’s Moral Judgment) (Grace et al., 2024, p. 251).

Derinda needs to provide a reasoning behind both why she thinks of palliative sedation as being akin to killing and in how her sense of integrity might be shaken by participating. Derinda will need to describe why and how providing palliative sedation would undermine her sense of self and integrity. For example, she needs to demonstrate how intractable suffering of a patient challenges her beliefs in supporting life at all costs. As a result, Derinda’s reasoning process may clarify her understanding of how her religious beliefs and values inform her view of her role as a nurse.

If on reflection Derinda is confident that it is in her best interests not to participate, then a plan must be made about how to grant this CO in a way that ensures the patient’s needs are met and that is least disruptive to all. A balance of benefit to the patient over harms to Derinda may need to be explored. There is evidence to suggest that unrelieved moral distress can cause physical and psychological harms and alter the person’s ability to engage with patients. In such instances, patient care is affected either by nurses distancing themselves from future emotional engagement with patients or nurse attrition (Rushton & Boss, 2013). Certainly, Derinda has a right to object, and be supported in this, if she is convinced her sense of integrity is threatened and her explanations are reasonable. Alternatives need to be identified to ensure that patient preferences are honored and plans put in place to prevent other such situations in the future. A key element of this process is to ensure non-abandonment of the patient and the avoidance of unintended harm caused by disparities in skill or preparation of other staff. It is especially important that this patient receives care at a minimum from an equally qualified nurse. For the charge nurse or evaluator as well as Derinda herself the potential consequences of a CO claim need to be made evident and understood. Thus, determining the validity of the CO claim is essential.

3. What strategies and resources are available to assist the charge nurse in exploring this particular situation where Derinda is considering a CO? (Rest’s Moral Judgement) (Grace et al., 2024, p. 251).

Regardless of whether Derinda’s hesitancy is due to feeling unprepared or is the result of an authentic CO, the charge nurse is faced with managing the situation in a way that reduces harms, provides the most benefit to those involved and does so in a fair, just manner. In the moment, this may be simply rearranging patient assignments for the day. However, there are likely to be ongoing consequences that must eventually be managed. For example, the emotional burden of caring for patients with palliative sedation may disproportionately fall to a smaller group of staff. Ideally, staff willing to provide the requested treatments would be able to do so voluntarily and without coercion. Without transparent and open dialogue about how to remedy the situation, Derinda may be the recipient of anger from her colleagues or feel isolated in her team. Proactively addressing these possibilities and creating open spaces for dialogue are essential elements of staff support. Reference to an existing CO policy would be helpful, but if none exists then this situation should provide an impetus for policy development as discussed later.

- Including Derinda in planning for a resolution for this CO claim.

The charge nurse will need to determine whether these are the only sorts of situations in which Derinda objects. If her CO is more extensive, for example, Derinda refrains from giving adequate pain medicines to relieve the suffering of those imminently dying but not on a palliative sedation regimen, then potential solutions may include her transferal to another unit altogether. Although pain and exacerbated pain are some of the most common symptoms in persons at the end of life (Deandrea et al., 2014), palliative sedation is not a very frequent procedure (Ferrell & Paice, 2019; Garetto et al., 2018). Alternatively, Derinda may continue to work in the unit but not be assigned to patients who are undergoing palliative sedation if there is sufficient staffing and advance planning to accommodate her request. A plan would then be required for those times when other nurses are not available to relieve Derinda from caring for a patient requesting palliative sedation.

b) Ethical assistance.

In many larger hospitals, resources such as ethics consultants are available. Where such resources exist, CO claim evaluators can benefit from accessing this resource both in dealing with the emergent situation and planning policy. Those with skills of ethical analysis can help provide clarity about hidden aspects of a situation and help identify the risks and benefits. When the concern is based on religious beliefs, hospital chaplains may be helpful to the person claiming CO. Similarly, after the fact, opportunities for nurses to engage in ethics discussions, ethics rounds as well as self and group reflections are important preventive ethics strategies. Grace and colleagues (2024) recommend assisting nurses to “reconcile… values, (and) core beliefs, with the priority of patient expressed desires” (p. 253).

4. What are Derinda’s, the charge nurse’s, and the institution’s responsibilities to ensure that a patient has their needs met despite the CO? (Rest’s Moral Motivation and Moral Character) (Grace et al., 2024, p. 251).

This question helps CO evaluators to deliberate the potential consequences of the CO claim for the respective situation, the personnel and patients involved, and the organization itself. Point 7 of the National Consensus Project for Quality Palliative Care proposes that “the goal of palliative care is the relief of physical, psychological, emotional, and spiritual suffering of patients and families” (Ferrell & Paice, 2019, p. 6). The responsibility of such a unit is to provide patients with the necessary care to relieve pain and other symptoms. Palliative sedation is considered in situations where patients are experiencing refractory pain and constitutes a usual and standard but infrequent practice in palliative care settings (Ferrell & Paice, 2019, p. 116). Although Derinda may still object to assisting in the procedure of palliative sedation, the CO needs to be reviewed carefully by drawing on existing evidence, policies as well as considerations on therapeutic and power relationships. Doing so in a transparent equitable manner builds trust among the healthcare team and the people they serve (Rushton & Reina, 2024).

To care for patients, nurses develop a therapeutic relationship. This relationship is characterized by the nurse’s knowledge of the patient, the professional stance, and competence for building a trustful, reliable, and constructive connection with the respective patients and their families (Erikson & Davies, 2017; Ferrell & Paice, 2019; Zulueta Egea et al., 2023). Nevertheless, power imbalance exists between nurses’ knowledge and skills, and patient need. Therefore, nurses must account for situations where power imbalance influences patient care and take care that they are listening to the patient, understanding their perspectives, and clarifying the boundaries of their personal and professional boundaries (Hart, 2015; Okpala, 2021).

5. What institutional or leadership support is available both the charge nurse and Derinda with respect to CO? (Rest’s Moral Judgment and Moral Character applied to leadership) (Grace et al., 2024, p. 251).

International, national, and institutional codes, guidelines and policies exist concerning CO (ANA American Nurses Association, 2025; ICN International Council of Nurses, 2021; Schouppe, 2018; Wicclair, 2014). Institutional policies should contain strategies for balancing respect for a nurse’s CO, while insuring safe, quality patient care (Wicclair, 2014). Should such policies not yet exist, all of the following are potential resources for such development: The EENCO (Grace et al., 2024), professional codes of ethics (e.g., ICN International Council of Nurse, 2021), the ANA position statement on risks and responsibility (ANA Center for Ethics and Human Rights, 2022), and bioethics literature related to CO (e.g., Wicclair, 2011).

- The ANA position statement and institutional policies on CO.

The position statement on *Risk and Responsibility in Providing Nursing Care* offers guidance for both Derinda and the institution for addressing CO in this case (ANA Center for Ethics and Human Rights, 2022). It is stated that “Conscience-based refusals to participate exclude personal preference, prejudice, bias, convenience, or arbitrariness” (ANA Center for Ethics and Human Rights, 2022, pp. 1–2). Therefore, Derinda must demonstrate that her CO claim is truly grounded in her deep-seated values. Her charge nurse, in contrast, must explore Derinda’s motives openly to get to the heart of the claim. The ANA statement also maintains that “Acts of conscientious objection may be acts of moral courage and may not insulate nurses from formal or informal consequences. Such refusal should be made known as soon as possible, in advance and in time for alternate arrangements to be made for patient care” (ANA Center for Ethics and Human Rights, 2022, p. 2). For Derinda’s case, this statement indicates the importance of a CO claim. Grounds for not accepting the CO claim must be well-founded. However, both ANA statements underscore that ensuring patient care is paramount. After all, nurses have to provide non-discriminatory care to patients, all the while preserving their own physical, psychological, emotional, and spiritual integrity (ANA Center for Ethics and Human Rights, 2022, pp. 1–2).

b) National and international policies.

Case 1 occurs in the USA. Therefore, the ANA’s (2025) Code of Ethics for Nurses with interpretive statements provides guidance on professional obligations to which all US nurses are bound. Additionally, the ANA’s position statement on *Risk and Responsibility in Providing Nursing Care* demands that “healthcare organizations should have in place policies and procedures which address such items as: conscientious objection… resource allocation, etc. Nurses should be able to communicate any identified risks through the appropriate organizational channels so adequate safeguards can be initiated” (ANA Center for Ethics and Human Rights, 2022, p. 3). Therefore, the charge nurse has a role in initiating policy development on CO for this particular unit, if not institution. Guidelines and standards differ among countries and it is incumbent on evaluators and policy developers to understand what safeguards exist in their region and/or country.

c) Preventive ethics.

Preventive ethics is the idea that emerging problems can sometimes be anticipated and strategies developed for their early resolution (Epstein, 2012). Important to preventive ethics related to a CO is an understanding of the organization’s mission and values and any CO policies that exist. Newly hired personnel should be made aware of the institution’s stance along with procedures for requesting a CO. Additionally, for units where COs are more likely to be invoked, patient care expectations and responsibilities should be described in detail to new hires, and their perspective sought on whether the unit is an appropriate place for them. The existence of preventive ethics strategies is unlikely to eliminate all cases of CO. In Derinda’s case, she may not have been aware of how she would actually feel in this case. Thus, informal evaluation of her CO should focus on understanding rather than being adversarial toward the person claiming CO (Wicclair, 2014, p. 267).

d) Provide safe space for reflection.

Another institutional strategy to anticipate and/or support COs is to provide the opportunity and a safe place for nurses to reflect on their beliefs, values, biases and prejudices together or individually. HCP with ethics expertise can offer guidance and education. For units where a CO might be expected or has occurred previously, educational opportunities need to be implemented for managers, nurses, and HCPs to work through their stance and to develop ways of accommodating the professional needs within the interprofessional team (Ewashen et al., 2013; Pakkanen et al., 2022).

### Case 2 - Conscientious Objection as Conscientious Commitment

At the beginning of the COVID-19 pandemic, one of the main countermeasures to its spread and adopted in many countries, Switzerland included (home of one of the authors), was a general lockdown (FOPH Federal Office of Public Health, 2013). In Switzerland, this measure was based on a legal act, namely the Swiss law on epidemics (FOPH Federal Office of Public Health, 2013). The law asserts the powers of the Swiss government to take protective measures against the spread of pandemics such as closing public transport systems, closing restaurants, and protecting healthcare institutions by restricting access to nurses and HCPs only. In the hospitals, this law translated into forbidding employees from taking vacations, being closed to family visits, and other such rules. Sometimes, healthcare institutional leadership mandated nurses to continue their work despite being sick themselves and sometimes threatening to fire them for noncompliance. During this time, sick and even dying patients were separated from their families. The restrictive visiting measures contributed to ethical challenges for patients, families, and bedside nurses (Miralles et al., 2021; Nguyen et al., 2021; Ortoleva Bucher et al., 2023; Sudai, 2021). For example, patients were physically alone at the time of death as their close family members were not allowed to support them in person. For these families, there was a danger of missing out on the last moments of the loved-one’s life and the subsequent memories. Additionally, there was anguish about patients’ isolated suffering and families not being able to provide comfort for them (Chen et al., 2021; Collingridge Moore et al., 2024; Sudai, 2021). In order to remind hospital and nursing home leadership of the potentially difficult consequence of this restrictive implementation of the policy, the Swiss Academy of Medical Sciences published an appeal to the leaders in healthcare (Ackermann et al., 2020) without noticeable effect. While strategies for avoiding the more serious consequences of isolation were proposed, implementation of these measures did not and has not yet occurred systematically.

Case 2 exemplifies a conflict of ethical commitment, a type of CO. It focuses on the case of Robert, married to Joanne and father of their two young children who had contracted COVID-19 and was hospitalized. His health deteriorated rapidly over a week leading to the need for mechanical ventilation. In turn he could no longer communicate directly with Joanne by phone, and she had to resort to calling staff for updates. Taking daily calls from this Joanne exacerbated the already overburdened staff and increased their distress as they had no positive progress they could report. Moreover, they had to reiterate that family was not allowed to visit because of the governmental COVID-19 protocols. Despite six weeks of treatment, it became obvious that Robert’s death was imminent. The nurses sought a way to allow Joanne and the children to see Robert, knowing that this was legally risky and might lead to their dismissal. The nurses decided that Joanne could enter the institution via a back corridor so she could see him via a window, and in this way say goodbye. For Joanne and the children this visit was a relief as much as it was horrific to see Robert in such a compromised state. The nurses bonded together over this event, telling no one for fear of reprisals. They felt that they had an ethical obligation to lessen the harms to this family caused by being isolated from one another. However, they felt like criminals and suffered ongoing distress (Bergman et al., 2020; O’Mathúna et al., 2023).

#### Analysis of Case 2

In contrast to case 1, case 2 involves the CO of a group of nurses. The CO is not voiced clearly to the leadership but is hidden and can be considered subversive. This is just one example of many similar situations. The task in such a case is to understand and manage fairly the balance of benefits and harms. We will draw on the EENCO’s five main questions to explore and analyze the ethical status of this case. A starting point is to consider the nurses’ reasons for choosing to go against the legal rules that prohibited visitation.

1. How does this group of nurses and their allied colleagues understand their professional responsibility and its limits? *(Rest’s Moral Sensitivity)* (Grace et al., 2024, p. 251).

The pandemic situation, and subsequent measures put into place to contain it, was a novel contemporary event. While everyone was affected in some way, it was particularly difficult for nurses, HCPs, and their patients who were suffering from serious COVID-19 symptoms. The decision of this group of nurses to organize clandestine visits was deliberate with the intention to provide ethical care of patients and their families. The balance was between prioritizing the needs of and the risks to individuals versus the collective needs of society for containment of the virus. The novel status of the situation was increased by lack of knowledge about the virus and its spread, and healthcare systems’ failure to adequately prepare for such a pandemic, which had been predicted (Clark et al., 2022).

These nurses determined that organizing an opportunity for family visits could be enacted while also minimizing risk of COVID-19 cross contamination. While the developers of the pandemic rules had good intentions, these rules also disadvantaged many (McMillan et al., 2021). The CO claim, then, can be reframed as one of a commitment to ethically grounded patient care in the face of rules or laws that do or did not serve patients well.

- Epidemic laws.

Drawing on an understanding of epidemics, and similar to actions taken in many countries (Miralles et al., 2021; Nguyen et al., 2021; Ortoleva Bucher et al., 2023; Sudai, 2021), Switzerland’s first response to COVID-19 included a general lockdown between March and May 2020. The lockdown and several other measures affected health service delivery very much as well as nurses, HCP, patients, and families. In hindsight, these responses by the Swiss government have been critically reviewed and considered questionable (Balthasar et al., 2022). The sequelae of these measures such as trauma and grief in patients, families, nurses, and HCP alike are now considered too severe. There are recommendations to modify these measures in future pandemic situations to limit fallout and negative sequelae (Balthasar et al., 2022; Clark et al., 2022; Monteverde & Gallagher, 2020). In case 2, the nurses were torn between the patient’s impending death, the provision of dignified care including the presence of Joanne and the children at the end of life; the grieving family who had not seen or spoken to Robert in weeks, the lockdown, and the restrictive rules in place in the institution. These values are central to the nurses’ code of ethics (ICN International Council of Nurse, 2021).

For the nurses’ group, Robert dying alone without Joanne and the children present was considered inhumane and going against the standards of a dignified end of life. From the nurses’ perspective, it was their professional duty to provide some means of connection for this family. In that, the nurses’ group manifested their obligation to advocate for patients and patient groups, a core HCP competency according to the CanMEDs reference framework, which guides Swiss healthcare practice (Zumstein-Shaha & Grace, 2023; Herion et al., 2019; Sottas, 2011). From a legal perspective, the nurses’ behavior cannot be condoned. However, it can be argued that the nurses were aware of all the dangers associated with the clandestine visit, particularly the problem of spreading the disease. They took measures to avoid any potential infection pathway such as providing personal protection to the family members and themselves. They made sure that the family only saw Robert through a window from a surrounding corridor without personal contact. While laws exist to serve the general case and institute societal protections, they do not always serve their intended purpose. Thus, decisions must sometimes be made about when and under which conditions it may be ethically supportable to flout an existing law.

b) Codes of ethics.

Codes of ethics for HCP are the given profession’s conceptualization of what might be expected from its members. They generally outline the responsibilities of the members of the professional group to individuals and society. For nurses internationally, the ICN code of ethics (2021) is informed by insights from nurses in more than 130 countries. The ICN (2021) maintains that “1.1 Nurses’ primary professional responsibility is to people requiring nursing care and services now or in the future,” (p. 9) and “1.8 Nurses demonstrate professional values such as respect, justice, responsiveness, caring, compassion, empathy, trustworthiness, and integrity. They support and respect the dignity and universal rights of all people, including patients, colleagues, and families.” (p. 9). Therefore, the rigorous policies in place during COVID-19 limiting family access to patients, particularly at the end of life, goes against both the stipulations of these two codes of ethics.

While it might be argued that one should work toward changing policies, this is a slow process. In the COVID-19 pandemic, a few were making policies for the majority with little recourse for immediate change. The group of nurses here faced a dilemma: allowing for an untenable situation for Robert, Joanne and the children, and themselves, or working toward solutions that would support the patient and his family despite “breaking the law” (FOPH Federal Office of Public Health, 2013) and violating hastily developed institutional policies. This is an example of a clash between what is ethical and what is legal. In view of these codes of ethics, the nurses’ rationale of going against the prevailing legal rules can be understood, if not accepted.

c) Institutional policies.

The hospital where this situation occurred had specific rules to uphold the Swiss laws in place during the pandemic. Mostly, these rules were hastily developed and did not fully consider all potential consequences of rigid implementation of the Swiss laws into healthcare settings. Research has since revealed that many institutions had their own interpretation of the Swiss laws (Balthasar et al., 2022; Ortoleva Bucher et al., 2023) or elsewhere (Sudai, 2021). Therefore, nurses organizing the family’s visit to see Robert could be viewed as an understandable exception to the rule. Exceptions to general rules require explicit and ethically grounded reasons to support them.

2. What does it mean for this group of nurses to say a conviction is held strongly enough that the likely harms to the objector outweigh the likely good for the patient? *(Rest’s Moral Judgment)* (Grace et al., 2024, p. 251).

By breaking the imposed law, nurses helped the family see Robert for a last time, if only through a window. Potentially, these nurses endangered other patients and HCP through exposure to a deadly virus by having this family visiting. Also, nurses created inequities for other patients and families in similar situations by only helping this family. Nevertheless, the nurses’ conviction to facilitate the visit despite the risks was strong, built on values of humanity and ethical responsibility. In the end, the clandestine visit remained unnoticed by the leaders of the unit and the hospital. However, the nurses continued to feel like criminals as the COVID-19 rules continued to be exercised rigorously.

- Codes of ethics.

Consistent with ICN Code of Ethics (2021), the patient is the primary obligation for nurses. Such values are the normative basis of nursing. It is important in this sort of situation that possible broader harms to the society are considered and that efforts to reduce harms are undertaken. The nurses followed their ethical obligations towards Robert and his family. They orchestrated the visit with minimum risk to them and all other patients in the unit. The visiting family did not have contact with any other patients or HCP, apart from the supportive nurses’ group. This way, potential contamination was reduced to a minimum. Considering the balance of good over harm is important but difficult, especially in cases such as the COVID-19 pandemic when knowledge is evolving.

3. What strategies and resources are available to the nurses’ group to assist in exploring this situation where the nurses are claiming CO? *(Rest’s Moral Judgment)* (Grace et al., 2024, p. 251).

As in case 1, similar resources and strategies are referred to in this case.

- National and international codes of ethics.

The ICN code of ethics (2021) supports that nurses can claim CO where their professional, ethical and moral values are endangered. Similarly, the recommendations by the American Thoracic Society (Lewis-Newby et al., 2015) encompass several occasions when CO can be accommodated. Among these are: “To protect clinicians’ moral integrity, to respect clinicians’ autonomy, to improve the quality of medical care, and to identify needed changes in professional norms and practices.” (Lewis-Newby et al., 2015, p. 221). These guidelines can help institutions, leaders, and individual HCP to better determine occasions for CO but also to explore the depths of the CO claim. Other international or national codes of ethics can be consulted to provide guidance. The EENCO (Grace et al., 2024), as is suggested here, offers a structured approach to explore each CO claim from all perspectives. Thus, arguments for or against it can be compiled and a balanced decision obtained.

b) Ethics and other literature.

The implementation of the Swiss law into institutional rules could have been more scrutinized by HCPs in general and respective leaders in healthcare. Ethics and other literature could have been consulted (McMillan et al., 2021). In this case, the consultation of guidelines of end-of-life care would have revealed that dignified care involves flexible visiting hours for Robert and his family whenever possible (Kesecioglu et al., 2024).

In nursing ethics literature, e.g., the concept of ‘moral courage’ has been described (Numminen et al., 2016; Pajakoski et al., 2021, 2024). Based on this concept, the respective leadership of that unit could have developed a more adequate application of rules by respecting the moral obligation of HCP of prioritizing patients’ needs, or by respecting the need for HCP to act in such a challenging situation.

Similarly, consulting contemporary ethics literature and ethical thinking can help one to be aware of successful strategies in place elsewhere. Seminal works such as the latest edition of Beauchamps and Childress’s Principles of Biomedical Ethics (2019), are a resource for describing and clarifying what is at stake, perhaps especially when the interests of individuals are pitted against the interests of the collective. Joining with others who have similar concerns can enhance the power to change rules that are not serving persons well. Other perspectives on ethics such as the ethics of care (Held, 2005) and theories of social justice (Powers & Faden, 2008) provide important perspectives.

c) HCPs ethics education.

In Switzerland, similar to other countries, the ICN code of ethics for nurses (2021) is taught in basic education leading to the nursing diploma and accreditation, i.e., at the Bachelor’s level or similar. However, how well professionals generally understand their ethical responsibilities towards individuals and society remains unclear (Milliken & Grace, 2015; Singh et al., 2016). Where codes of ethics exist, HCPs are responsible for understanding their warrants. A strong underlying principle is prioritizing the care of individuals within a HCPs purview, while also accounting for the public good. The nurses’ group, here, manifests an understanding of their responsibilities by prioritizing Robert and his family over the legal and institutional rules in place during the pandemic, while also trying to control COVID-19 transmission.

d) Ethics consultations.

Consultation with ethics experts where available can help parse out the nuances of a situation, the potential risks of various actions as well as benefits. Thus, the evaluation of the CO claim is conducted with a more objective lens. This may help to represent each perspective in a balanced way so that all parties concerned are treated equal.

4. What are the nurses’ group and the institution’s responsibilities to ensure that a patient has their needs met in spite of the CO? (*Rest’s Moral Motivation and Moral Implementation and Character)* (Grace et al., 2024, p. 251).

In this situation, while some patients may have their needs met, others will not and still others may be put at additional risk. These inequities in accommodation can create additional ethical conflicts especially if the process is not applied equally to all patients. However, social justice may require that any inequalities that exist are slanted toward benefiting the least well off (Rawls, 1971).

- Procedure for addressing CO claims.

A paucity of recommendations exists as to how to proceed with proposing a CO or conscientious commitment of this nature or how to address it. The framework for deciding this should ideally be part of an institutional policy or at the very least, the institution should consult with existing norms such as the codes of ethics (e.g., ICN International Council of Nurse, 2021) or other policies such as the paragraph on freedom of conscience in the Swiss constitution (Schweizerische Eidgenossenschaft [Swiss Confederation], 1999, pp., Art. 15), or, e.g., the American Thoracic Society’s statement on CO (Lewis-Newby et al., 2015).

The ANA (ANA Center for Ethics and Human Rights, 2022) goes one step further and states that institutions have a responsibility for developing, implementing, and regularly revising policies on CO based on evolving information. After all, healthcare institutions have the responsibility of finding ways to improve patient care while limiting cross-contamination and creating systematic processes to address unintended consequences in a timely manner. If that had been the case here, the nurses’ group might have chosen to voice their concerns in a more adequate fashion than organizing clandestine visits. There is also an ethical responsibility of the institution to learn from prior situations and anticipate future needs. This requires a systematic process for quality improvement and accountable action plans.

In this case, the nurses’ group voiced their concerns to unit leaders prior to acting but did not receive assistance or support. Therefore, the nurses creatively circumvented the problem, which in itself can raise ethical issues (Bianchi & Ghirotto, 2022; Debono et al., 2013). While the good for Robert and his family is at least partially resolved, the problem for other such patients is not. Moreover, such actions pose a risk to all involved that should be carefully considered before proceeding. Hence, this type of strategy can only be a temporary solution (Bianchi & Ghirotto, 2022; Debono et al., 2013). Eventually, HCPs must try to advocate for policy change based on a clear delineation of the ethical conflict and suggest ways that good care can be provided. Being able to describe the pros and cons of the problem in ethical terms, providing support from codes of ethics, and bringing other resources to bear could add persuasive strength to the process and permit initiation of a systemic evaluation.

b) Safe spaces to reconsider one’s values.

The government mandated restrictions in response to the pandemic, as well as their translation into institutional policies contributed to moral distress and likely burnout of nurses and HCP (Rushton et al., 2021). Clearly, a global pandemic created both expected and unexpected ethical challenges for nurses and the people they serve. Despite the Swiss law (FOPH Federal Office of Public Health, 2013), institutions should be proactive in addressing foreseeable situations such as these. At least, institutions should engage in identifying ways for nurses and other healthcare professionals to provide humanized care to patients and their families without legal or criminal consequences. Creating forums that are psychologically safe, without fear of retaliation, offers HCP opportunities to examine and process the moral distress or injury they experience when confronted with ethically challenging situations (Morley et al., 2022).

5. What institutional or leadership support is available for this group of nurses with respect to CO? *(Rest’s Moral Motivation and Moral Implementation and Character applied to leadership)* (Grace et al., 2024, p. 251).

Exemplified here is the issue of a group of nurses’ objection to unethical policies and the need for institutional and leadership support to provide good care in novel circumstances. The nurses’ group here resorted to workarounds. A workaround occurs when an existing policy or protocol is seen as not meeting the needs of patients, yet is not amenable to quick or easy revision. This strategy carries positive as well as negative sequelae and only short-term solutions to a long-term problem (Bianchi & Ghirotto, 2022; Debono et al., 2013).

- International and national policies.

In Switzerland, the predominant policy in place during the pandemic was the respective law. Drawing on this law, each healthcare institution basically established their own policy or rule concerning the lockdown, patient and family visits, maintaining social distance, and so on. In the beginning of the pandemic, many healthcare institutions were very restrictive concerning family visits of patients evidencing the fear of transmission (Bergman et al., 2020; Ortoleva Bucher et al., 2023). The longer the pandemic continued, this fear was alleviated as transmission pathways emerged (Balthasar et al., 2022). However, in many cases, healthcare institutions continued with their restrictive policies, thereby contributing to unnecessary trauma and other sequelae in patients, families as well as HCPs (Monteverde & Gallagher, 2020; Ortoleva Bucher et al., 2023). While written policies related to CO may not always be available internationally, as in case 2 (Arbeitsgruppe Rechte des medizinischen Personals [Working Group for Rights of Medical Personnel], 2002), international guidelines are helpful such as the ICN code of ethics (2021).

b) Collaboration and cooperation.

Historically, nurses and other HCPs have sometimes found themselves stymied from meeting their professional goals by institutional rules. The goals of healthcare institutions and HCPs differ in many ways. Significantly, the institution must focus on managing resources and being cost-effective to stay viable and provide services. While HCPs maintain a focus on providing humane services that provide the good that the profession exists to serve, health, wellbeing, relief of suffering, etc., a balance must be struck. Without HCPs the institution cannot function. Without the institution HCPs may not have the resources they need to provide a ‘good’ for patients. Therefore, it is beneficial to both entities to collaborate.

c) Providing safe space for reflection.

As in case 1, the protagonists of case 2 could have profited from a safe space had it been available. The nurses’ group could have discussed the case within the whole team and maybe even with the leadership openly without fear of repercussions. Clarifying the values and beliefs that exist among the HCP of one unit in a healthcare institution is an important step into creating a collaborative environment (CIHC Canadian Health Collaborative, 2024). At the very least, the guiding values and beliefs in that unit would have been clarified and potential conflicts could have been identified preventively.

## Discussion

Conscientious Objection in healthcare occurs in various settings and circumstances. The claim may be to maintain one’s own sense of integrity or to redress a problem that is affecting or likely to affect patients. The EENCO (Grace et al., 2024) can be a helpful evaluation framework for nurses, leaders, other HCPs, and regulators as well as to orientate policy development. The emphasis is on professional responsibilities to provide the good to the patient that the profession exists to provide. Responsibilities of institutional leadership are highlighted. Nurses and HCP have a mandate to prioritize patient care. In the case of CO, this mandate must be upheld, all the while the claimant is supported to reflect on their reasons, and in the exercise of their objection, provided it is determined reasonable and sustained after a period of guided reflection. A valid CO must be more than an expression of an opinion or bias. Rather, the claimants’ general underlying belief system and its conclusions must be transparent to evaluators and others who will be affected (Fry-Bowers, 2020; Wicclair, 2017). Ongoing opportunities to explore such cases in ‘ethics rounds’ can also be helpful in debriefing and raising consciousness about recurring problems. When available, other ethics resources such as an ethics consultant, are helpful.

## Insights from EENCO for Policy Development

Institutional and regulatory policy is critical for evaluating an actual or potential CO as illustrated in the two cases. Some countries and institutions have policies about CO, while others do not. Also, some countries have overarching guidelines, while others possess certain laws (ANA Center for Ethics and Human Rights, 2022; Arbeitsgruppe Rechte des medizinischen Personals [Working Group for Rights of Medical Personnel], 2002; Fry-Bowers, 2020). On an international level, no other practical guideline or framework specific to the broad spectrum of claims of CO in healthcare settings was found; thus the need to develop one such as EENCO (see Table 1). The purpose of a policy is the establishment of a process and criteria for persons to request exemption from participating in an aspect of patient care or treatment, based on a values conflict. It lays out the responsibilities and accountabilities of involved parties: the person objecting, supervisors and leaders, the institution.

- Limitations.

For the purpose of demonstrating the application of the EENCO, two specific cases were selected deliberately. These two cases do not necessarily represent the whole range of potential situations leading to CO in nurses and other HCP. However, case 1 is representative of a classic CO scenario, whereas case 2 represents doing good over harm, because of perceived inhumane institutional rules before being recognized as involving a CO to harmful protocols. Thanks to the EENCO, both cases could be treated similarly and explored from all angles. Evidence was included in the subsequent arguments.

## Recommendations and Conclusions

The application of the (EENCO) framework can help expose hidden elements and nuances in a proposed or actual CO by nurses and healthcare professionals, thereby illuminating strategies that can lessen associated harms. Since CO can take various forms, it requires careful exploration to balance benefits and burdens, guiding ethical decision-making in clinical conflicts. As a rule, the balance of benefits over burdens guides ethical decision-making and actions in most clinical conflicts. However, calculating the balance can be challenging due to power gradients between providers and recipients. Claimants must ensure their claims are rooted in reason and consider the impact on others. Alternatively, flouting harmful rules or taking action to highlight unethical practices should be ethically analyzed. While immediate actions may be necessary to provide patient care, addressing widespread harm requires a higher-level approach. The EENCO provides a structured framework for addressing such situations by exposing underlying values, supporting individuals, and exposing existing policies. Applying the EENCO to cases highlighted international and national healthcare CO policies, providing helpful resources. It also presented core items for institutional CO policies. Additionally, an existing CO policy guides leaders and claimants.

Signs: 65’646.

**Table 1:** Components of institutional policy related to conscientious objection

EENCO Applied to Policy Development	Essentials of CO Policies as the Relate to the Following Entities: HCP healthcare providers C inter- and intradisciplinary colleagues L leaders/supervisors I institution
Moral Sensitivity (HCP, I, L) Discerning the nature of a CO and its attendant consequences for all involved.	• Definition of CO what it is (authentic concern) and what it is not (arbitrary dislikes, biases, and prejudices). With examples (e.g., palliative sedation, abortion, unethical practices that can harm patients). • Definitions of the types of actions involved. (e.g., participation, performing, assisting, referral). • Expectations that the objector has reflected on their CO, its meaning to them and others who may be involved or affected. • Examples of resources/supports and strategies to use in reflecting.
Moral Judgment (HCP, L) Appreciation of professional responsibilities to use knowledge and skills for patient good. To prioritize the patient’s needs over one’s own. Understanding one’s accountability to patient and colleagues. Process of balancing goods over harms to all involved.	• The role of professional responsibilities to prioritize patient good over one’s own except in justifiable and justified circumstances (codes of ethics, fiduciary nature of professional responsibility). • Statement about the sorts of considerations and justifications that may be used to support the CO. • What literature has been drawn upon to formulate the policy. • What sanctions are possible or probable. • Timeframes for making an objection known. • How the burdens for patient, colleagues and the institution will be managed. • Legal obligations to ensure patient safety (EMTALA, etc.).
Moral Motivation (HCP, C, L) The cognitive and emotive aspects that lead one to act. The impetus to act based on a determination of best action in the situation.	• How the CO will be supported and/or sanctioned. • Possible accommodations that can be made. • Statement of the responsibility of an objector to consider working in a different type of environment if recurrence of CO is likely.
Moral Character (HCP, L, C. I) Possession of the social and psychological skills and strength of character necessary to carry out the chosen action. The cognitive and emotive aspects that lead one to persevere in the action and overcome presenting obstacles	• Statement about nurses’ duty to self and patients (obligations to act against CO when a patient or colleague is endangered). • What education and support might be needed • What resources will the institution provide • How will COs be tracked. • Chain of reporting

## Declarations

### AI

No generative AI was included.
